# Hepatic Rupture in HELLP Syndrome: Report of Two Patients and a Review of Peripartum Surgical Care and Transfusion

**DOI:** 10.1002/ccr3.70059

**Published:** 2025-01-24

**Authors:** Sarah N. Larson, Trevor F. Killeen, Laura Bowman, Sruthi Shankar, Emily Stock, Lindsay Welton, James V. Harmon

**Affiliations:** ^1^ University of Minnesota Medical School ‐ Twin Cities Minneapolis Minnesota US; ^2^ Department of Surgery University of Minnesota Minneapolis Minnesota US

**Keywords:** general surgery, HELLP syndrome, hepatic rupture, massive transfusion, preeclampsia

## Abstract

Herein, we report the cases of two patients with hemolysis, elevated liver enzymes, and low platelets syndrome who underwent emergent Cesarean sections that were complicated by massive hemorrhage due to undiagnosed hepatic rupture. Intraoperative General Surgery team intervention, early activation of massive transfusion protocol, hemostatic resuscitation, and transfer to ICU resulted in the survival of both patients.

## Graphical abstract

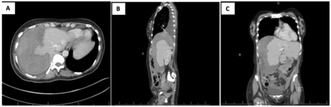

## 1 Introduction

Preeclampsia is characterized by the development of new‐onset hypertension with proteinuria and other laboratory abnormalities after 20 weeks of pregnancy [1]. Hemolysis, elevated liver enzymes, and low platelets (HELLP) syndrome affects 0.1%–0.6% of all pregnancies and 4%–12% of pregnancies complicated by preeclampsia [1]. HELLP syndrome most commonly presents between 27 and 36 weeks of gestation; however, it can also occur in the early postpartum period [1]. Both preeclampsia and HELLP syndrome have been hypothesized to share a similar pathophysiology, namely arising from similar placental structural abnormalities that induce compensatory responses to the state of hypoxia that occurs [1, 2]. These changes promote abnormal coagulation and vasospasm along with increased maternal systemic vascular resistance [1, 3]. Subcapsular liver hematoma is an especially rare comorbidity that has been estimated to complicate the cases of < 2% of patients diagnosed with HELLP syndrome [1, 4, 5]. Hepatic rupture can be fatal, mainly due to rapid blood loss from hepatic hemorrhage, which has been reported to result from increased sinusoidal fibrin deposition and coagulopathy associated with HELLP syndrome [4, 6]. Sinusoidal obstruction due to thrombosis can result in subcapsular hematomas that may remain stable or later rupture. Fetal demise in pregnancies with hepatic rupture reportedly occurs in more than 30% of cases [6]. Unfortunately, development of subcapsular liver hematoma or hepatic rupture is unlikely to be detected on routine peripartum ultrasound exams based on current practice patterns. However, the use of ultrasonography for a patient presenting in extremis may lead to a potential diagnosis [7, 8].

Herein, we report the cases of two patients with HELLP syndrome who underwent emergent Cesarean sections that were complicated by massive hemorrhage due to undiagnosed hepatic rupture.

## 2 Case Presentations

Case 1: A 32‐year‐old G1P0 at 27 weeks’ and 3 days’ gestation was admitted to the hospital for preeclampsia with severe right upper quadrant abdominal pain. The patient was hypertensive (171/85 mmHg), hyperreflexic with one beat of clonus, and exhibited trace pretibial edema. On laboratory evaluation, she was found to have 1+ proteinuria, anemia (hemoglobin, 9.6 g/dL), thrombocytopenia (94,000 platelets/μL), elevated liver enzymes (alanine transaminase [ALT], 241 U/L; aspartate aminotransferase [AST], 175 U/L), and elevated lactate dehydrogenase (LDH 198 U/L). The patient was diagnosed with preeclampsia with severe features complicated by HELLP syndrome. Intravenous magnesium and betamethasone were administered for maternal seizure prophylaxis and fetal neurologic and lung maturity in anticipation of imminent delivery. The patient proceeded with an urgent primary classical Cesarean section through a Pfannenstiel incision. Upon entry to the abdomen, hemoperitoneum secondary to hepatic rupture was encountered. There was no evidence of uterine bleeding. The General Surgery team was urgently consulted intraoperatively, and massive transfusion protocol (MTP) was activated. A live‐born female was delivered, followed by an intact placenta. Uterine tone was adequate following intravenous pitocin administration and uterine massage. Vertical hysterotomy was reapproximated using double‐layer suture closure with excellent hemostasis.

The General Surgery team explored the abdomen through the Pfannenstiel incision. Ongoing hepatic hemorrhage from a ruptured right‐sided subcapsular hematoma was controlled with abdominal packing and application of multiple surface hemostatic agents, including Arixtra (GlaxoSmithKline, Triangle Park, NC), Surgicel (Baxter International Inc., Deerfield, IL), and Surgiflo (Johnson & Johnson, New Brunswick, NJ). Intravenous tranexamic acid (1 g intravenous [IV] push) was administered to stabilize the clot. The intra‐abdominal cavity was irrigated with sterile normal saline, and a #19‐round Blake drain was placed overlying the hepatic hematoma. The fascia and skin were reapproximated. Two units of packed red blood cells (pRBC) and one unit of fresh frozen plasma (FFP) were administered as part of MTP during delivery and surgery.

The estimated intraoperative blood loss as recorded in the operative report was 1800 mL.

**FIGURE 1:**
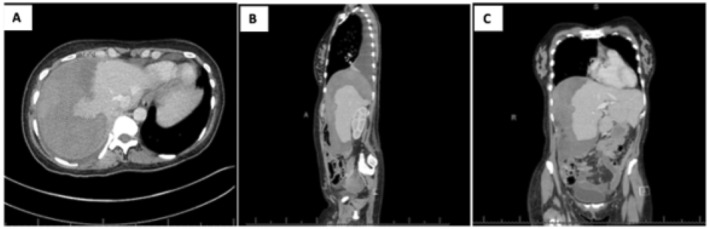
(A–C) Patient in Case 1, postoperative day 9. Abdominal CT scans demonstrate a perihepatic hematoma measuring 26 × 14 × 11 cm along with a large right pleural effusion. 1A. Transverse view; 1B. Sagittal view; 1C. Coronal view.

The patient was transferred to the Surgical Intensive Care Unit (SICU) where elevated systemic blood pressure and significant tachycardia were managed using IV nifedipine and metoprolol. Serial hemoglobin/hematocrit was tracked with the patient's hemoglobin remaining stable without need for additional transfusions. Thrombocytopenia and elevated liver function tests (LFTs) improved following resuscitation. Serum LDH remained elevated (457 U/L) at the time of discharge. These laboratory values confirmed the diagnosis of complete HELLP syndrome using the Tennessee criteria. The patient was transferred to the maternity ward on postoperative day (POD) 2. The patient was discharged on POD 7 but readmitted on POD 9 with new right‐sided flank pain and dyspnea. A right pleural effusion was identified on computed tomography (CT) imaging, and 500 mL of a dark transudative fluid was percutaneously drained. The perihepatic hematoma appeared stable on abdominal CT (26 × 14 × 11 cm) and ultrasonography (21 × 15 × 11 cm). On POD 11, the patient was discharged home (Figure 1). Follow‐up with the General Surgery clinic confirmed complete clinical resolution of symptoms and perihepatic hematoma using abdominal ultrasonography. The patient had no postoperative complication during a 2‐year follow‐up period. Unfortunately, while there is no indication of the neonate's condition in the maternal electronic record, it is our understanding that the baby has survived to normal childhood.

**FIGURE 2:**
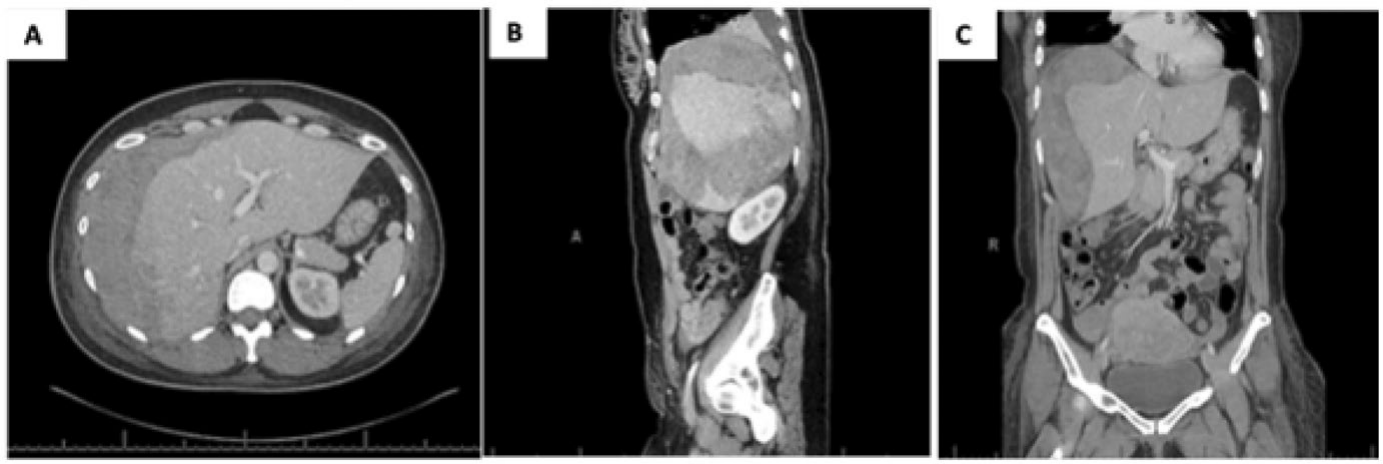
(A–C) Patient in Case 2. Abdominal CT scan on POD 5 demonstrated a large hepatic hematoma measuring 21 × 11 × 9 cm along with areas of hypoattenuation in segments 4–8, possibly representing areas of hepatic infarction. 2A. Transverse view. 2B. Sagittal view. 2C. Coronal view.

**FIGURE 3:**
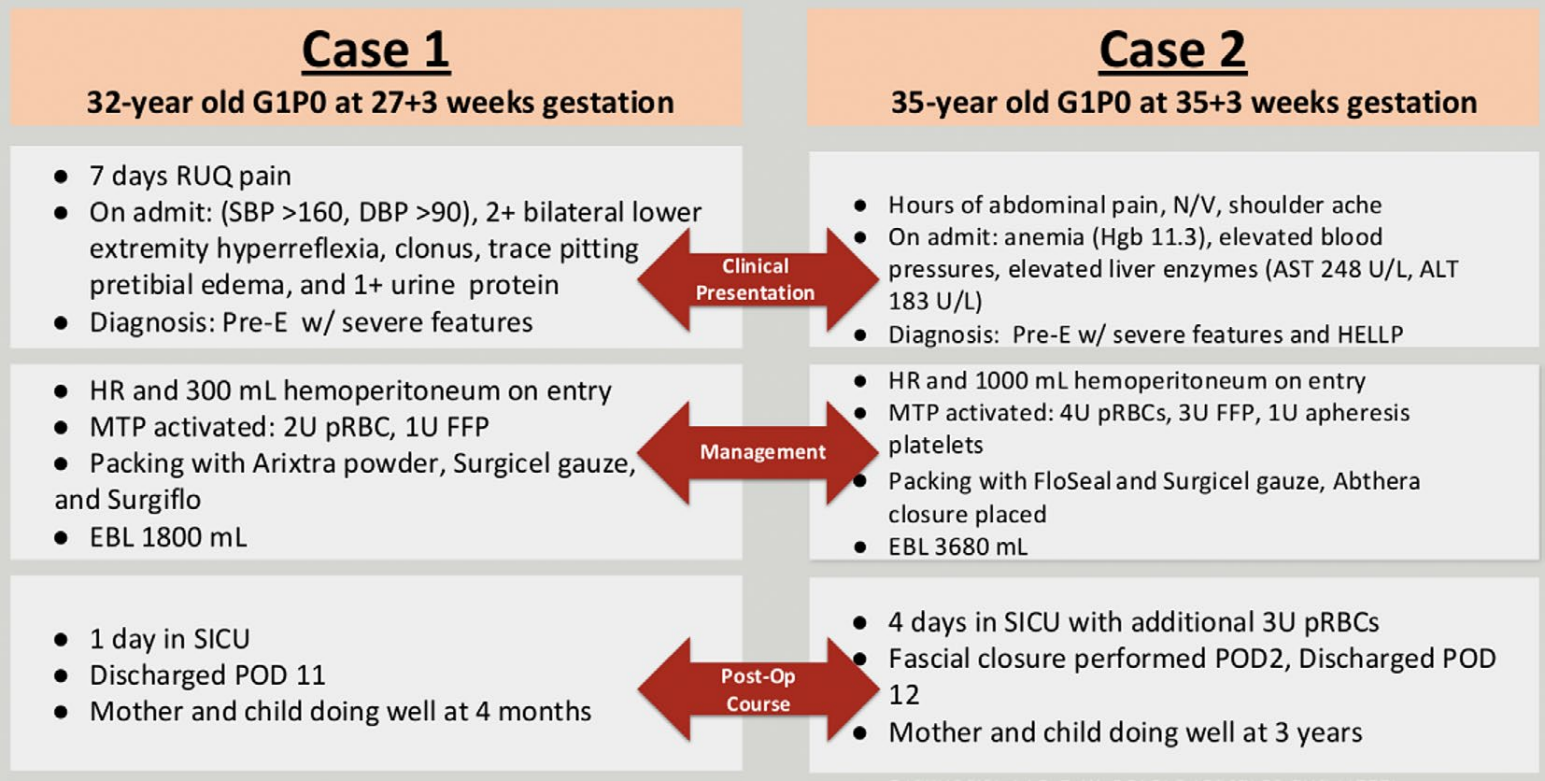
Comparison of Cases 1 and 2. AST, Aspartate transaminase; ALT, alanine aminotransferase; DBP, diastolic blood pressure; EBL, estimated blood loss; FFP, fresh frozen plasma; HELLP, hemolysis, elevated liver enzymes, and low platelets; Hgb, hemoglobin; HR, hepatic rupture; MTP, massive transfusion protocol; N/V, nausea and vomiting; POD, postoperative day; pRBC, packed red blood cells; preE, preeclampsia; RUQ, right upper quadrant; SBP, systolic blood pressure; and SICU, surgical intensive care unit.

Case 2: A 35‐year‐old G1P0 at 35 weeks’ and 3 days’ gestation was admitted for preeclampsia with severe features complicated by HELLP syndrome. The patient presented with seven hours of abdominal pain which radiated to the right shoulder with associated nausea and vomiting. The patient's comorbidities included a congenital bicuspid aortic valve and gestational diabetes. On admission, she was hypertensive (143/84 mmHg), anemic (hemoglobin 11.3 g/dL), and thrombocytopenic (38,000 platelets/μL), with a notable transaminitis (AST, 248 U/L; ALT, 183 U/L). Intravenous magnesium and betamethasone were administered for maternal seizure prophylaxis and fetal neurologic and lung maturity in anticipation of imminent delivery. Fetal heart monitoring revealed a nonreassuring fetal status. During emergent primary low transverse Cesarean section, hemoperitoneum associated with hepatic rupture was noted. A pale and limp, live‐born male was delivered and transferred to the neonatal intensive care team. The placenta showed evidence of abruption. The patient was hypotensive, and MTP was initiated. Uterine atony and hemorrhage were managed using uterotonics (Pitocin and Hemabate), uterine massage, and placement of B‐Lynch and O'Leary sutures. The General Surgery team was urgently consulted intraoperatively. The patient required transfusion of four units of pRBCs, three units of FFP, and three apheresis units of platelets in the operating room. Ongoing hepatic hemorrhage from a ruptured right‐sided hepatic hematoma was controlled with abdominal packing and application of multiple surface hemostatic agents, including FloSeal (Baxter International Inc., Deerfield, IL) and Surgicel Gauze (Johnson & Johnson, New Brunswick, NJ). The abdomen was irrigated with sterile normal saline, two #19 round Blake drains were placed, and a temporary abdominal dressing (AbThera, Acelity, San Antonio, TX, USA) was applied. Estimated blood loss was 3600 mL including 1000 mL hemoperitoneum on entry from the obstetrics portion and 100 mL from the general surgery portion. The patient remained intubated and sedated and was transferred to the SIC with an open abdomen U, where an additional three units of pRBC, one unit of FFP, and one unit of platelets were transfused. On POD 2, the patient was taken back to the OR for repeat exploration at which time there was no evidence of further bleeding and the fascia was closed in a routine fashion. Serial hemoglobin/hematocrit was tracked postoperatively with the patient's hemoglobin and platelets remaining stable without need for additional transfusions. The patient was transferred to the maternity ward on POD 3. Copious salmon‐colored incisional drainage was observed on POD 5, and abdominal CT revealed a hepatic hematoma (21 × 11 × 9 cm), along with a 1.3‐cm fascial defect that was managed nonoperatively (Figure 2). On POD 5, the patient was dyspneic. Examination revealed decreased breath sounds, and chest radiography confirmed bilateral pleural effusions. Hyperkalemia responded to IV furosemide administration. Hypocalcemia, presumably caused by MTP, was corrected following IV calcium replacement. LFTs and thrombocytopenia improved following resuscitation. On POD 12, the patient was discharged home and had no postoperative complications during a 5‐year follow‐up period. Unfortunately, while there is no indication of the neonate's condition in the maternal electronic record, again it is our understanding that the baby has survived to normal childhood. Figure 3 contrasts the two case reports.

## 3 Methods

We completed a retrospective chart review of two patients with HELLP syndrome and hepatic rupture. Both patients signed a written consent form for the publication of the case material. The study did not require review from the Institutional Review Board (IRB) for case report publication at our institution.

We also performed a literature review using the OVID Medline from January 2009 to October 2021. Search terms used included: “ruptured subcapsular hematoma,” “HELLP syndrome,” and “hepatic rupture.” We limited our search to articles published in English. A total of 44 patient reports met our analysis criteria [9, 10, 11, 12, 13, 14, 15, 16, 17, 18, 19, 20, 21, 22, 23, 24, 25, 26, 27, 28, 29, 30, 31, 32]. Thereafter, we focused on the 13 patient reports that permitted analysis of required blood transfusions. The following criteria were used to select 13 case reports for our review of perioperative blood transfusion in HELLP syndrome‐associated hepatic rupture: the report included clinical and laboratory diagnoses of HELLP syndrome, the patient underwent peripartum surgical management of hepatic rupture, and the report detailed the type and quantity of blood components transfused. Eighteen variables were analyzed using RStudio (R‐Foundation, Boston, MA).

## 4 Conclusion and Results

Herein, we report two cases of peripartum hepatic rupture caused by HELLP syndrome. MTP activation resulted in hemostatic resuscitation in both patients. Both patients were successfully managed using MTP and hemostatic techniques at the time of delivery. Due to the high maternal mortality rate from ruptured liver hematoma and diagnostic challenges, early activation of MTP is of paramount importance when a pregnant patient with HELLP syndrome presents with hemodynamic instability. The symptoms of HELLP syndrome resolved completely and did not have further consequences, as demonstrated in the two patient reports. Having MTP reserves available intraoperatively during a catastrophic hemorrhage helps avoid resuscitation failure.

Maternal mortality was reported in four of 44 patients (9%), fetal loss in 15 of 44 patients (34%), intact subcapsular hematoma in six of 44 patients (14%), and hepatic rupture in 36 of 44 patients (82%). The estimated mean blood loss was 3000 mL. The mean ICU stay was 7.3 days (+/− 5.8 days), and mean hospital stay was 19 days (+/− 15 days) (Table 1). Interventional Radiology embolization was performed in 11 of 44 patients (25%), surgical intervention in 35 of 44 patients (80%), hepatic resection in two of 44 patients (5%), and liver transplantation in one of 44 patients (2.3%). The mean and standard deviation of transfused blood components in the 13 patients are as follows: pRBC, nine units (+/− 10 units); FFP, ten units (+/− 7.6 units); and platelets, four apheresis units (+/− 2.6 units) (Table 2).

**TABLE 1:** Patient characteristics and treatment outcomes for 44 patients [7, 8, 9, 10, 11, 12, 13, 14, 15, 16, 17, 18, 19, 20, 21, 22, 23, 24, 25, 26, 27, 28, 29, 30, 31] and comparison with 13 patients with hepatic rupture in HELLP syndrome who underwent surgery and were transfused blood products.

	Analysis of all 44 cases of HR or SCH in PreE or HELLP syndrome	Select analysis of 13 studies where blood products were quantified and surgery was performed to manage HR
Maternal death (*n*)	4 (9.1%)	1 (7.7%)
Fetal death (*n*)	15 (34.1%)	7 (53.8%)
Both maternal and fetal death (*n*)	4 (9.1%)	1 (7.7%)
Mean age (years)	30.8	31.9
Mean gestational age (weeks)	32.3	30.9
Method of HR diagnosis	Imaging (47.7%) vs intra op (52.3%)	Imaging (46.2%) vs intra op (53.8%)
Time at HR diagnosis (intraoperative, preoperative, postoperative)	Intra (40.9%), post (11.4%), pre (38.6%)	Intra (38.5%), post (23.1%), pre (38.5%)
Received blood (n)/Mean EBL	15	13
Mean EBL (mL)	3000	3325
HD vs ICU days (mean ± SD)	18.1 +/− 12.2 vs. 10.33 +/− 10.2	18.8 +/− 14.6 vs. 7.33 +/− 5.77

Abbreviations: EBL, estimated blood loss; HD, hospital day; HELLP, hemolysis, elevated live enzymes, and low platelets; HR, Hepatic rupture; ICU, intensive care unit; PreE, preeclampsia; SCH, subcapsular hematoma.

**TABLE 2:** Data on the amount of blood products administered to 13 patients in select analysis [7, 8, 9, 10, 11, 12, 13, 14, 15, 16, 17, 18, 19, 20, 21, 22, 23, 24, 25, 26, 27, 28, 29, 30, 31].

Reported clinical attributes	Reported clinical outcomes
RBC (unit average +/− SD)	9 +/− 10
FFP (unit average +/− SD)	10 +/− 7
Apheresis platelets (unit average +/− SD)	4 +/− 2.6
Received all three product types	7/13
Estimated blood loss range (mL)	0–12,000
ICU admission	11/13

Abbreviations: FFP, fresh frozen plasma; ICU, intensive care unit; RBC, red blood cell.

## 5 Discussion

We reviewed 44 published case reports, of which 13 included the type and quantity of blood components transfused, permitting an analysis of transfusion requirements. Although early activation of MTP is the standard practice for managing hemorrhage in general, it is particularly crucial in the management of maternal hemorrhage [15]. Pressurized administration of warmed blood products facilitates hemostatic resuscitation. At our facility, laboratory assays are conducted every hour during MTP, including hemoglobin, platelet counts, international normalized ratio (INR), partial thromboplastin time (PTT), fibrinogen, thromboelastography (TEG), and Rotational thromboelastometry (Werfen, Bedford, MA). We have also instituted a restricted two‐way phone system with the blood transfusion cooler to optimize communication between the bedside team and blood bank personnel.

Limited reports, including a smaller number of patients, have reported maternal mortality rates ranging from 18% to 86%, and fetal demise rates ranging from 31% to 80% [4]. Gupta et al. published a review of 45 patient reports in 2021, demonstrating a maternal mortality rate of 15% and a fetal mortality rate of 41% [11]. In comparison, our literature review demonstrated a 9% maternal mortality and a 34% fetal mortality rate. Our findings were in concordance with those of Jeffries et al. [11]

In rare instances, hepatic rupture in HELLP syndrome may be managed non‐surgically with hepatic artery embolization and reversal of coagulopathy; however, emergency surgical intervention is most often required to control hemorrhage [13]. Early activation of MTP provides the required hematological and resuscitative support. Although we did not extend the Cesarean incision in these two cases, extension of the incision may be required for adequate exposure and control of hemorrhage. Packing the right upper abdomen with laparotomy pads, followed by application of surface hemostatic agents, often achieves hemostasis. Some patients may present with both uterine hemorrhage and hepatic rupture, as was exemplified in Case 2. Hepatic artery embolization can be performed intraoperatively or postoperatively as an additional technique [33]. Moreover, cross‐clamping of the aorta or deployment of resuscitative endovascular balloon occlusion of the aorta are possible salvage maneuvers [34]. Hypotensive, hypothermic, and coagulopathic patients may benefit from damage‐control surgery and temporary abdominal closure, as was performed in Case 2. Returning to the OR within 6–72 h often permits additional hematoma evacuation, abdominal washout, possible surgical drain placement, and fascial closure [5].

Early diagnosis and timely intervention are indicated for hepatic rupture. However, due to the significant overlap of clinical symptoms at the time of delivery, the diagnosis of hepatic rupture can be challenging [30]. Several authors advocate the use of imaging in clinically stable patients to detect subcapsular liver hematoma prior to rupture [5, 6, 7]. However, there is no universal agreement regarding the use of imaging modalities as first line for diagnosis. Kaltofen et al. advocated the use of ultrasonography as first‐line imaging followed by magnetic resonance imaging (MRI) as a standard protocol to detect subcapsular liver hematoma in obstetric patients with upper abdominal pain [5]. They proposed this plan due to the fact that approximately half of all patients with HELLP syndrome demonstrate abnormal but nonspecific liver findings on imaging [5]. Ultrasonography is inexpensive and accessible, and it can detect hemoperitoneum by enabling the visualization of free abdominal fluid. However, Singh et al. recommended the use of ultrasonography to detect subcapsular hematomas in stable symptomatic patients, highlighting the risk of accidental rupture from hematoma compression by the ultrasound probe [4]. Westbrook et al. proposed that CT or MRI is ideal for investigation [6]. Although CT and MRI enable better image quality, they are costly and require additional time for imaging, which may not be practical for patients who are clinically decompensating. In both our patients, a ruptured subcapsular hematoma was discovered intraoperatively at the time of surgical delivery without the use of pre‐operative imaging, and CT and ultrasound were used for postoperative monitoring of subcapsular hematoma resolution.

## Author Contributions

**S.N.L., T.F.K., L.B., S.S.:** data collection, data analysis, literature review, and drafting of the manuscript. **E.S.:** drafting and editing of the manuscript. **L.W.:** literature review, data analysis, and drafting and editing of the manuscript. **J.V.H.:** project conceptualization and drafting and editing of the manuscript.

## Ethics Statement

The University of Minnesota Institutional Review Board does not require IRB approval for case reports or literature review.

## Consent

Signed consent for publication of both case reports is available upon reasonable request from the author.

## Conflicts of Interest

The authors declare no conflicts of interest.

## Permission to Reproduce Material From Other Sources

No materials from other sources were utilized in the creation of this manuscript.

## Data Availability

The case report details in the medical record cannot be released; however, our spreadsheet pertaining to the literature review can be made available upon reasonable request from the author.
